# Unusual Presentation of Popliteal Cyst on Magnetic Resonance Imaging

**DOI:** 10.1155/2016/1214030

**Published:** 2016-11-23

**Authors:** Tsuyoshi Ohishi, Masaaki Takahashi, Daisuke Suzuki, Yukihiro Matsuyama

**Affiliations:** ^1^Department of Orthopaedic Surgery, Enshu Hospital, Hamamatsu, Shizuoka 430-0929, Japan; ^2^Joint Center, Jyuzen Memorial Hospital, Hamamatsu, Shizuoka 434-0042, Japan; ^3^Department of Orthopaedic Surgery, Hamamatsu University School of Medicine, Hamamatsu, Shizuoka 431-3192, Japan

## Abstract

Popliteal cyst commonly presents as an ellipsoid mass with uniform low signal intensity on T1-weighted magnetic resonance images and high signal intensity on T2-weighted images. Here, we describe a popliteal cyst with unusual appearance on magnetic resonance imaging, including heterogeneous intermediate signal intensity on T2-weighted images. Arthroscopic cyst decompression revealed that the cyst was filled with necrotic synovial villi, indicative of rheumatoid arthritis. Arthroscopic enlargement of unidirectional valvular slits with synovectomy was useful for the final diagnosis and treatment.

## 1. Introduction

Popliteal cyst, or Baker's cyst, is commonly seen in patients with rheumatoid arthritis [1]. It is easily differentiated from other cystic or solid tumours according to its appearance on magnetic resonance imaging (MRI) [2]. Here, we present a case of popliteal cyst in a patient with rheumatoid arthritis that had an unusual appearance on MRI. Arthroscopic enlargement of unidirectional valvular slits was useful for the final diagnosis and treatment.

## 2. Case Report

A 74-year-old man was referred to our hospital with a 2-month history of a painful mass in the popliteal fossa of the right knee. The patient had undergone conservative treatment for a diagnosis of osteoarthritis of the right knee for the previous 4 months at a nearby clinic. The patient was 158 cm tall and weighed 58 kg and demonstrated a limp due to right knee pain. He had emphysema and carcinoma of the stomach, both of which were well controlled.

**Figure 1:**
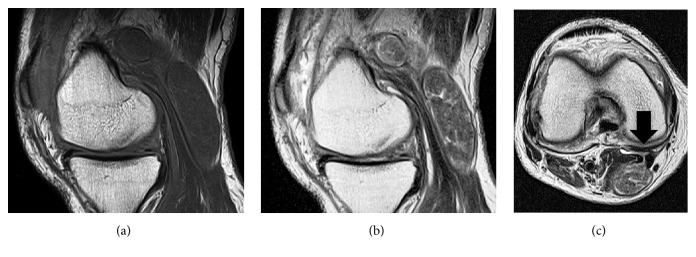
Preoperative sagittal T1-weighted (a) and T2-weighted (b) and axial T2-weighted (c) magnetic resonance images. A cyst was detected with low signal intensity on T1-weighted images and heterogeneous intermediate signal intensity on T2-weighted images. A connecting path was seen between the cyst and the subgastrocnemius bursa (arrow), compatible with the characteristics of popliteal cyst.

On physical examination of the right knee, an elastic soft mass measuring 5 cm × 3 cm with a smooth surface was palpable in the popliteal fossa. The mass was tender on palpation without redness or warmth. Range of motion was −10° of extension and 120° of flexion due to contracture. Tenderness was elicited on the medial joint line. McMurray's test was positive with pain on the medial joint line, but a clicking sound was not elicited. Patellar ballottement also was positive. No anteroposterior or lateral instability was observed. Laboratory data revealed a C-reactive protein level of 1.26 mg/dL and a haemoglobin level of 12.9 mg/dL but were otherwise within normal range. Blood examinations pertaining to rheumatoid arthritis were not performed at the initial visit. Plain radiographs of the right knee showed grade 2 medial compartment osteoarthritis. MRI revealed a well-defined popliteal mass with low signal intensity on T1-weighted images and heterogeneous intermediate signal intensity on T2-weighted images (Figures 1(a) and 1(b)). The cyst connected to the subgastrocnemius bursa through a path between the medial head of the gastrocnemius muscle and the semimembranosus tendon (Figure 1(c)). The provisional diagnosis was popliteal cyst with some solid contents inside the cyst in an osteoarthritic knee.

**Figure 2:**
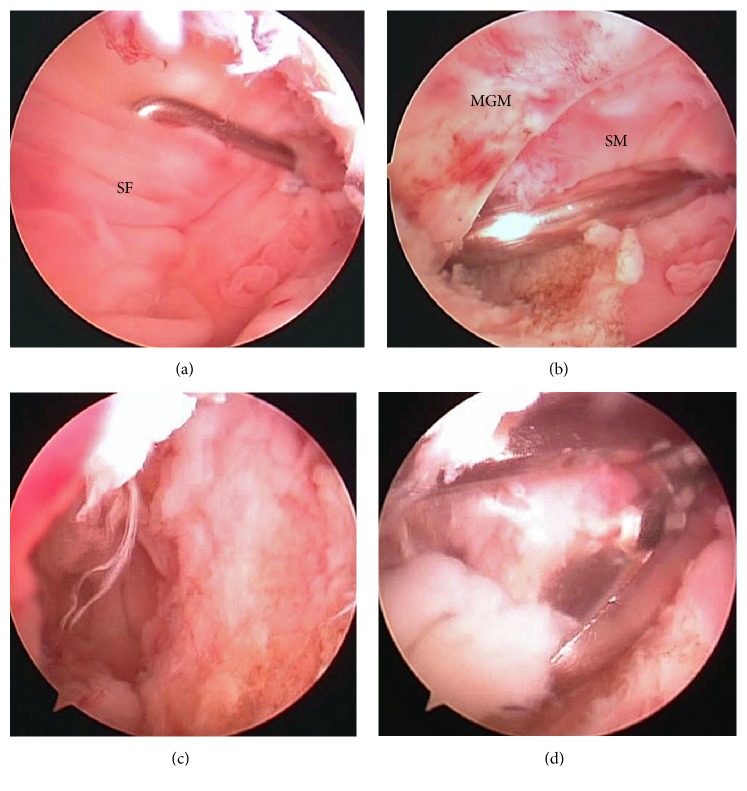
Arthroscopic views from the posterolateral portal through the transseptal portal. (a) The synovial fold (SF) was identified using a probe introduced from the posteromedial portal. (b) After removing the synovial fold, a switching rod was inserted between the medial head of the gastrocnemius muscle (MGM) and the semimembranosus muscle (SM), which was the orifice of the popliteal cyst. (c) The orifice of the popliteal cyst was enlarged by resecting the limited parts of the medial head of the gastrocnemius muscle and the semimembranosus muscle. (d) The material inside the cyst was pushed out from behind and removed using forceps.

**Figure 3:**
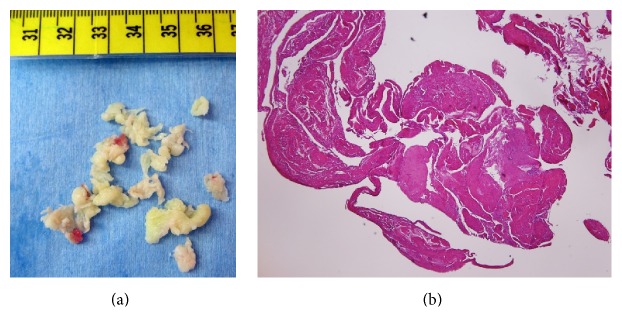
Photographs of gross (a) and histologic (magnification ×40) (b) findings of the material inside the cyst. Fragments of necrotic synovial villi with fibrinoid degeneration are observed.

Arthroscopic surgery under general anaesthesia was performed. Synovial proliferation was observed in the suprapatellar pouch and gutters of both sides, which were excised. To access the popliteal cyst, a transseptal portal was made after creating posteromedial and posterolateral portals using our technique [3]. Synovial proliferation also was observed in the posteromedial and posterolateral compartments. Arthroscopic cyst decompression was performed using our procedure [4]. First, the synovial fold was identified (Figure 2(a)) and removed using a motorized shaver from the posteromedial portal while viewing from the posterolateral portal via the transseptal portal. A vertical slit between the medial head of the gastrocnemius muscle and the semimembranosus tendon was identified (Figure 2(b)) and enlarged by destructing the unidirectional valvular slit (Figure 2(c)). Once the orifice of the cyst was enlarged, abundant yellowish synovia-like fragments were pushed out manually from behind until the popliteal mass was not palpable on the back (Figure 2(d)). Pathologic examination revealed that the synovial villi in the suprapatellar pouch were thickened with inflammatory cells, blood vessels, and fibrinoid-degenerated connective tissue, compatible with rheumatoid arthritis. The material inside the cyst included fragments of necrotic synovial villi with fibrinoid degeneration (Figure 3).

**Figure 4:**
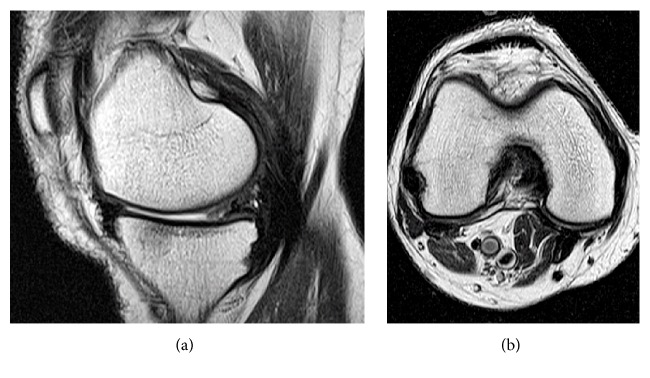
T2-weighted sagittal (a) and axial (b) magnetic resonance images taken 1 year postoperatively. No evidence of the popliteal cyst is observed.

Range of motion of the right knee and weight-bearing gait were allowed from 2 days postoperatively. Postoperative blood examinations revealed positive results for rheumatoid factor and an anticyclic citrullinated peptide antibody level of 78.1 U/mL. The final diagnosis was popliteal cyst filled with necrotic synovia accompanied by rheumatoid arthritis. No swelling, deformity, or tenderness was found in other joints, including the contralateral knee, fingers, elbows, or shoulders. Administration of methotrexate 6 mg/week was started after surgery. MRI performed 1 year postoperatively showed no evidence of the popliteal cyst (Figure 4). At 2 years postoperatively, recurrence of the cyst was not observed and the patient had no difficulty in performing activities of daily living.

## 3. Discussion

Popliteal cyst, or Baker's cyst, is caused by a one-way valvular mechanism of the slits between the medial head of the gastrocnemius muscle and the semimembranosus muscle [5]. On MRI, popliteal cyst commonly presents as an ellipsoid mass with uniform low signal intensity on T1-weighted images and high signal intensity on T2-weighted images [2]. The connection between the cyst and the subgastrocnemius bursa also can be detected on axial MRI. In our case, the ellipsoid shape with connection between the cyst and the subgastrocnemius bursa was compatible with popliteal cyst, but the signal intensities inside the cyst were unusual. The popliteal cyst in this case showed low signal intensity on T1-weighted images and heterogeneous intermediate signal intensity on T2-weighted images. Thus far, pigmented villonodular synovitis, hematoma, and infection have been reported in cases of popliteal cyst with unusual appearance on MRI [6, 7]. Unless the connection between the cyst and the subgastrocnemius bursa can be identified on MRI, malignant tumour, aneurysm, or benign solid tumour should be considered [8]. In all previous cases with unusual signal intensities inside the popliteal cyst, open resection was performed for diagnosis and treatment [6, 7]. The unusual presentation of the popliteal cyst on preoperative MRI was due to necrotic synovial villi inside the cyst. A one-way valvular mechanism between the cyst and the joint could have been responsible for the accumulation and concentration of synovial villi in the cyst, with proliferation in the joint due to rheumatoid arthritis.

In addition to rheumatoid arthritis, popliteal cysts can be caused by many other underlying pathologies [9]. Anatomically, a popliteal cyst can be divided into two categories. Primary cysts have no communication between joint and cyst and are predominantly found in children without joint disorders. In contrast, secondary cysts have a communication between joint and cyst through a one-way valve mechanism and are mostly observed in adolescents [10]. The majority of popliteal cysts are classified as secondary cysts. All pathologies that cause joint effusion may also cause a secondary popliteal cyst. Meniscal tears, ligament insufficiency, cartilage lesions, osteoarthritis, infectious arthritis, villonodular synovitis, and rheumatoid arthritis can cause a popliteal cyst [9]. Among these, rheumatoid arthritis is a well-known cause of popliteal cysts, since inflammation and synovial proliferation can destroy the unidirectional valve mechanism between the medial head of the gastrocnemius muscle and semimembranosus muscle [11].

Conservative therapy including antirheumatic drugs is the first choice of treatment for popliteal cyst with rheumatoid arthritis. However, we could not diagnose this patient with rheumatoid arthritis preoperatively. According to previous reports, open excision of popliteal cyst results in a high rate of recurrence [12]. Arthroscopic enlargement of the unidirectional valvular slit with or without cystectomy, which recently has become widely accepted, is less invasive and has a low rate of recurrence [4, 13]. In this case, arthroscopic surgery followed by administration of antirheumatic drugs was effective for the final diagnosis and treatment.
